# Enhancement of Minor Ginsenosides Contents and Antioxidant Capacity of American and Canadian Ginsengs (*Panax quinquefolius*) by Puffing

**DOI:** 10.3390/antiox8110527

**Published:** 2019-11-05

**Authors:** Min-Soo Kim, Sung-Joon Jeon, So Jung Youn, Hyungjae Lee, Young-Joon Park, Dae-Ok Kim, Byung-Yong Kim, Wooki Kim, Moo-Yeol Baik

**Affiliations:** 1 Department of Food Science and Biotechnology, Kyung Hee University, Yongin 17104, Korea; 2 Department of Food Engineering, Dankook University, Cheonan 31116, Korea; 3 Department of Science in Korean Medicine, Kyung Hee University, Seoul 02447, Korea

**Keywords:** antioxidant activity, ginsenosides, *Panax quinquefolius*, puffing

## Abstract

The effects of puffing on ginsenosides content and antioxidant activities of American and Canadian ginsengs, *Panax quinquefolius*, were investigated. American and Canadian ginsengs puffed at different pressures were extracted using 70% ethanol. Puffing formed a porous structure, inducing the efficient elution of internal compounds that resulted in significant increases in extraction yields and crude saponin content. The content of minor ginsenosides (Rg2, Rg3, compound K) increased with increasing puffing pressure, whereas that of major ginsenosides (Rg1, Re, Rf, Rb1, Rc, Rd) decreased, possibly due to their deglycosylation and pyrolysis. Furthermore, 2,2′-azino-bis (3-ethylbenzothiazoline-6-sulphonic acid) (ABTS) radical scavenging activity, total phenolic content, total flavonoid content, amount of Maillard reaction products, and acidic polysaccharides content increased with increasing puffing pressure, but 2,2-diphenyl-1-picrylhydrazyl (DPPH) radical scavenging activity did not. There was no substantial difference in the results between puffed American and Canadian ginsengs. Consequently, these results suggest that puffing can be a promising novel technology for processing *P. quinquefolius* to achieve higher levels of minor ginsenosides and obtain value-added products.

## 1. Introduction

*Panax quinquefolius* L. (American and Canadian ginsengs) is the main ginseng cultivar in the United States and Canada [1]. *P. quinquefolius* administration is known to be effective in recovering fatigue, improving immunity, and controlling blood pressure and cholesterol level [2,3,4]. These effects are mediated by ginsenosides, ginseng-specific saponin components [5]. Ginsenosides have a glycoside structure, and their biological effectiveness varies depending on their structure. In addition to ginsenosides, ginseng is known to contain other active ingredients such as acidic polysaccharides and phenolic compounds [6].

The ginsenosides of ginseng include Rb1, Rb2, Rc, Rd, Re, Rf, Rg1, Rg2, Rg3, Rh, Compound K, F1, and F2 [7,8]. Ginsenosides are divided into protopanaxadiols (PPD) and protopanaxatriols (PPT) depending on the presence of a hydroxyl group at C3 or C6 and the presence or absence of sugar [9]. Among them, ginsenosides Rb1, Re, Rg1, Rc, and Rd account for more than 70% of the total amount of *P. quinquefolius* ginsenosides [10]. The genus *Panax* includes two main species, *Panax ginseng* (Korean ginseng) and *P. quinquefolius* [11]. There are distinctive differences in ginsenoside content between *P. ginseng* and *P. quinquefolius*. Ginsenoside Rg1, Rb1, and Rf contents are high in *P. ginseng*, while *P. quinquefolius* contains low levels of them [12]. In general, *P. quinquefolius* is known to have higher amounts of ginsenoside Rb1 and Re [13]. It was reported that ginsenosides Rg2, Rg3, Rh1, and Rh2, specific components produced by thermal stimulation, showed preventive effects against cancer and inhibited cancer cell growth [14].

In Asia, there are two types of ginseng: white ginseng is produced by drying raw ginseng, and red ginseng is produced by steaming raw ginseng at 98–100°C for 2–3 h [15]. Red ginseng shows a larger number of pharmacological effects than white and raw ginseng [16]; in particular, antioxidant activity, antioxidant enzyme activity, and cell viability are high [15]. It was reported that the minor ginsenosides content of red ginseng is high [17], while these compounds are not found in white ginseng [18]. In 2015, ginseng was registered as an international food standard by CODEX (Codex Alimentarius International Food Standards). Changes in the content of ginsenosides and bioactive ingredients in ginsengs usually take a long time; therefore, red ginseng production and puffing may be useful to enhance the pharmacological properties of ginseng products. In the near future, processed ginseng products will be major items in the international functional food market.

To execute puffing, a unique food-processing method, pressure is suddenly lowered to atmospheric level from a high pressure in a high temperature regime. Puffing induces the gelatinization of starch and increases the volume of the material resulting from the evaporation of water [18]. Puffing occurs in two steps. After rapid heating at atmospheric pressure, the sudden evaporation of water causes a rapid decrease in pressure by transfer of superheated steam [19]. Puffing causes chemical and physical changes including starch gelatinization in cereal grains [20], ginsenoside profile changes in red ginseng [18], increase in antioxidant activity of white ginseng [21], denaturation and reorganization of proteins in ginseng [22], removal of water and formation of a porous structure in barley [23], and inactivation of enzymes that cause deterioration by lipid oxidation during storage of red ginseng [24]. *P. ginseng* has been mainly used in studies on the changes in ginsenoside content caused by puffing [18,25]. Puffing of herbal resources has been reported to the greatly increase antioxidant activities [18,21,24,25]. However, limited information is available on the effect of puffing on the ginsenosides and the bioactive components of *P. quinquefolius*. In this study, we thoroughly investigated the changes in ginsenosides content and antioxidant activity of American and Canadian ginsengs caused by puffing at different pressures.

## 2. Materials and Methods

### 2.1. Materials

Dried four-year-old commercial *P. quinquefolius* ginseng roots were purchased from a Wisconsin Ginseng Farm (Wisconsin, USA) and Rainey Ginseng Farms, LTD (Ontario, Canada) in 2014.

### 2.2. Chemicals

The compounds 2,2-diphenyl-1-picrylhydrazyl, 2,2′-azobis-(2-amidinopropane) dihydrochloride, 2,2′-azino-bis (3-ethylbenzothiazoline-6-sulphonic acid), Folin–Ciocalteu reagent, carbazole, galacturonic acid, gallic acid, and catechin were purchased from Sigma-Aldrich (St. Louis, MO, USA). Phosphate-buffered saline (PBS) was purchased from Welgene (Gyeongsan, Korea). Ascorbic acid was purchased from Reagent Duksan (Ansan-si, Republic of Korea). HPLC ginsenoside standards were purchased from Ambo Institute (Daejeon, Republic of Korea).

### 2.3. Puffing Process

To minimize the carbonization of ginseng at high temperature, dried ginseng and rice were mixed at a ratio of 1:4 (*w*/*w*). Dried ginseng (200 g) and rice (800 g) were placed in a rotary gun puffing machine (PPsori Co., Namyangju, Korea) and heated. When the gauge pressure reached 490 kPa, the medium pressure was released to 294 kPa, and then the rotary gun puffing machine was heated again. For subsequent measurements, when the pressure reached 686 kPa, 784 kPa, 882 kPa, and 980 kPa, the puffing machine door was opened to reach atmospheric pressure. Dried ginseng without puffing treatment was used as the control group.

### 2.4. Color Measurement

The color changes of puffed ginsengs were confirmed by a color difference meter (JC801, Color Techno System, Tokyo, Japan). Ginseng samples were finely ground with a blender and filtered through a 100-mesh sieve. The range of hunter L, a, and b value coordinates ranged from L = 0 (black) to 100 (white), a = −80 (greenness) to 100 (redness), and b = −80 (blueness) to 70 (yellowness). The colorimeter was calibrated with a standard plate of L = 98.26, a = 0.24, and b = −0.24 before measurement.

### 2.5. Extraction Yield

Five grams of puffed ginseng were ground and mixed with 125 mL of 70% ethanol (1:25, *w*/*v*), followed by stirring at room temperature for 30 min. The extracts were filtered through a funnel in a Kimble filtering flask using a Whatman No. 2 filter paper (Whatman, Maidstone, England). The extracts were dried in a hot-air dryer (HB-502M, Han Beak Scientific Co., Bucheon, Korea) at 105 °C, and the extraction yield was calculated using the following Equation (1):

(1) $$Extraction\;yield\;\left(\%\right)=\frac{\left(W_{2}-W_{1}\right)}{A}\times \frac{E}{E^{\prime }}\times 100$$

where

*W*_1_ = Weight of empty aluminum dish (g)
*W*_2_ = Weight of aluminum dish and solid (g)
*A* = Weight of dried ginseng (g)
*E* = Total volume of extract (mL)
*E′* = Used volume of extract (mL).

### 2.6. Crude Saponin Content

The crude saponin content of the extracts was determined by the diethyl ether method. Dried solids were mixed with distilled water to a total volume of 50 mL. Sample (5 mL), distilled water (20 mL), and diethyl ether (25 mL) were mixed in a separation funnel. The mixture was shaken well and allowed to stand for 30 min for the separation of the aqueous and the organic layers. The separated diethyl ether layer was discarded, and the remaining water layer was mixed with 25 mL of water-saturated *n*-butanol, followed by shaking and another 30 min of rest for further separation. The separated *n*-butanol layer was collected and washed with water-saturated *n*-butanol. This process was repeated three times. The collected *n*-butanol layer was concentrated using a rotary vacuum evaporator (N-11, EYELA, Tokyo, Japan) under reduced pressure. After concentration, the sample was dried in an oven at 105 °C for 2 h. The crude saponin content was calculated by weight changes using the following Equation (2):

(2) $$Crude\;saponin\;content\;\left(\frac{\mathrm{mg}}{g\;\mathrm{ginseng}}\right)=\frac{\left(W_{1}-W_{2}\right)}{W_{3}}\times \frac{A}{B}$$

where

*W*_1_ = Weight of the dried sample and flask (mg)
*W*_2_ = Weight of the flask (mg)
*W*_3_ = Weight of total dried ginseng (g)
*A* = Weight of total concentration (g)
*B* = Weight of used concentration (g).

### 2.7. Ginsenoside Profile

Ginsenoside profile was analyzed by an HPLC system by dissolving the crude saponin concentrates in 5 mL of HPLC-grade methanol, followed by filtering with a Millipore filter (pore size 0.45 μm). The instrument was a 1260 Infinity II LC system (Agilent, Santa Clara, USA) equipped with a Kinetex C18 column (50 × 4.6 mm, Phenomenex, CA, USA) and a UV detector at 203 nm. The binary gradient-elution solvents consisted of distilled water (mobile phase A) and acetonitrile (mobile phase B). The flow rate of the mobile phase was 0.6 mL/min, the sample injection volume was 5 μL, and the analytical column temperature was maintained at 45 °C. The following gradient elution procedure was used: 0–7 min, 81% A, 19% B; 7–14 min, 71% A, 29% B; 14–25 min, 60% A, 40% B; 25–28 min, 44% A, 56% B; 28–30 min, 30% A, 70% B; 30–31.5 min, 10% A, 90% B; 31.5–34 min, 10% A, 90% B; 34–34.5 min, 81% A, 19% B; 34.5–40 min, 81% A, 19% B.

### 2.8. Antioxidant Activity

#### 2.8.1. DPPH Radical Scavenging Activity

The 2,2-diphenyl-1-picrylhydrazyl (DPPH) radical scavenging activity of the extracts was measured with a modified version of the method of Blois (1958) [26] using 2,2-diphenyl-1-picrylhydrazyl and 80% methanol to prepare a 0.1 mM DPPH solution. The absorbance of the DPPH solution was adjusted to 0.650 ± 0.020 at 517 nm using 80% methanol. Extract (0.05 mL) was added to 2.95 mL of the adjusted DPPH solution, and the mixture was allowed to stand in a dark room at 23 °C for 30 min. Subsequently, the absorbance at 517 nm was measured. As standard and blank, ascorbic acid and distilled water were used, respectively. DPPH was calculated using the following Equation (3):

(3) $$\%\;inhibition=\frac{A_{reference}-A_{sample}}{A_{reference}}\times 100$$

where

A*_reference_* = absorbance of the blank
A*_sample_* = absorbance of the sample
A*_reference_* = mixture of 0.1 mL of 80% MeOH and 2.9 mL of DPPH radical solution.

#### 2.8.2. ABTS Radical Scavenging Activity

The 2,2′-azino-bis (3-ethylbenzothiazoline-6-sulphonic acid (ABTS) radical scavenging activity was determined according to the following procedure [27,28]. The solutions of 1.0 mM 2,2′-azobis-(2-amidinopropane) dihydrochloride and 2.5 mM 2,2′-azino-bis (3-ethylbenzothiazoline-6-sulphonic acid) were mixed in 100 mL PBS. The mixture was stirred at 70°C for 30 min and then cooled to room temperature. The solution was filtered using a 0.45 μm syringe filter and diluted with PBS to achieve an absorbance of 0.650 ± 0.020 at 734 nm. The radical solution was stored at 37 °C. Extract (20 μL) and radical solution (980 μL) were mixed and reacted at 37 °C for 10 min, and the absorbance was measured at 734 nm. As standard and blank, ascorbic acid and distilled water were used, respectively.

### 2.9. Total Phenolic Content

Total phenolic content (TPC) was determined using the following procedure with some modifications [29,30]. To 2.6 mL of deionized water, 200 μL of Folin and Ciocalteu’s phenol reagent and 200 μL of extract were added and mixed. After 6 min, 2 mL of 7% Na_2_CO_3_ was added and mixed. The mixture was allowed to react at 25 °C for 90 min, and the absorbance was measured at 750 nm. Gallic acid was used as a standard, and deionized water was used as a blank.

### 2.10. Total Flavonoid Content

Total flavonoid content (TFC) was determined using the following method [31]. Distilled water (3.2 mL) and sample (0.5 mL) were mixed, and 0.15 mL of 5% NaNO_2_ was added. After 5 min, 0.15 mL of 10% AlCl_3_ was added. After 1 min, 1 mL of 1 M NaOH was added, and the absorbance was measured at 510 nm. Catechin was used as a standard, and distilled water was used as a blank.

### 2.11. Acidic Polysaccharides

Degradation of acidic polysaccharides was measured using a colorimetric method [32]. Samples (5 mL) were mixed with 3 mL of distilled water and 0.25 mL of 0.1% carbazole and placed in a water bath at 85 °C for 5 min. After cooling for 20 min at room temperature, the absorbance was measured at 525 nm. Galacturonic acid was used as a standard, and distilled water was used as a blank.

### 2.12. Maillard Reaction Products

To measure the level of the Maillard reaction products (MRPs), the extracts were diluted 50-fold, and the absorbance was measured at 420 nm using a spectrophotometer.

### 2.13. Statistical Analysis

All experiments were repeated in triplicate. Experimental data were analyzed by analysis of variance (ANOVA) and expressed as mean ± standard deviation. A Duncan’s multiple range test was conducted to assess significant differences among experimental mean values (*p* < 0.05, 0.01, or 0.001). All statistical computations and analyses were conducted with SAS software (version 8.2, SAS Institute, Inc., Cary, NC, USA).

## 3. Results and Discussion

### 3.1. Morphology

**Figure 1:**
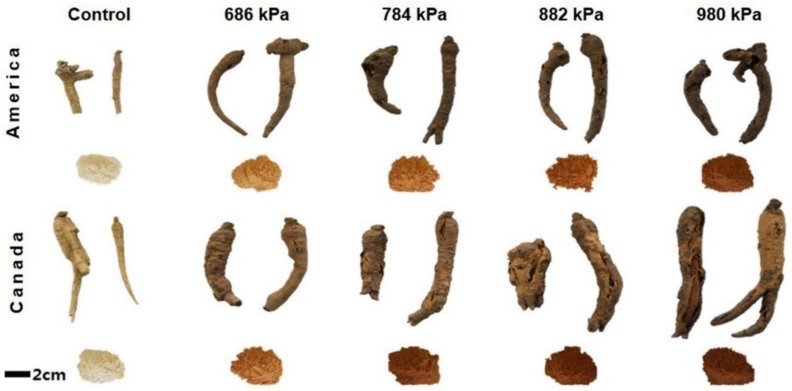
Morphology of puffed American and Canadian ginsengs.

The appearance of both types of ginseng according to puffing pressure is shown in Figure 1. Originally, Canadian ginseng was bigger than American ginseng before puffing, and regardless of the puffing pressure, the volume of Canadian ginseng was larger than that of American ginseng. It was reported previously that browning was further enhanced at high pressure in puffed red ginseng [18]. In particular, the surface was frayed starting at 784 kPa and separated from the interior at 882 kPa, resulting in the formation of blisters or air pockets inside the ginseng.

Puffing also changed the color of both ginsengs (Table 1). Regardless of the ginseng type, the L value decreased as the puffing pressure increased, suggesting increased pigmentation with puffing. The a and b values were higher than those of the control groups but decreased as the puffing pressure increased. This indicated that puffing increased the redness and yellowness of both ginsengs. On the other hand, the highest redness and yellowness values were observed at the lowest puffing pressure tested. The L value was consistent with the trend of MRPs content, which increased with increasing puffing pressure (Table 2). The higher were the temperature and pressure, the more active was the Maillard reaction between amino acids and sugars in ginseng [23,33].

### 3.2. Extraction Yield and Crude Saponin Content

The extraction yields and crude saponin content of puffed American and Canadian ginsengs are shown in Table 1. Before puffing, in the control group, both extraction yield and crude saponin content of Canadian ginseng were higher than those of American ginseng. Puffed American and Canadian ginsengs revealed higher extraction yields and crude saponin contents than those of the control groups. Puffing caused cell wall breakdown and porous structure formation, resulting in easy elution of active components from ginseng [18]. Although puffing increased both extraction yield and crude saponin content in American and Canadian ginsengs, no increasing trends in yield and content with increasing puffing pressure were found. The puffed American and Canadian ginsengs showed the highest extraction yield at 784 kPa and 880 kPa, respectively. In contrast, both ginsengs showed the highest crude saponin content at 686 kPa. Consequently, puffing increased both extraction yield and crude saponin content of each ginseng type at different puffing pressures.

### 3.3. Changes in Ginsenosides

**Figure 2:**
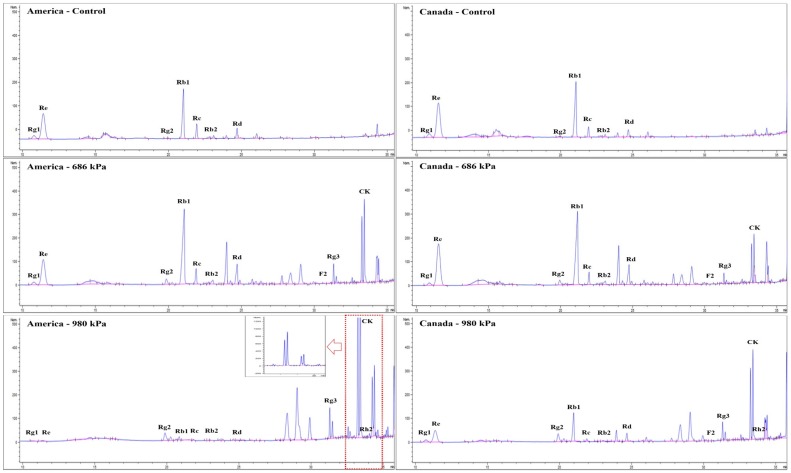
HPLC chromatograms of puffed American and Canadian ginsengs.

The HPLC chromatograms of puffed American and Canadian ginsengs are shown in Figure 2. In control groups of American and Canadian ginsengs, Rb1, Rb2, Rc, Rd, and Re were the major ginsenosides, all known to be characteristic of ginseng. In contrast, after puffing, the major ginsenoside content gradually decreased as puffing pressure increased in both American and Canadian ginsengs, resulting in the production and increase of minor ginsenosides such as Rg2, Rg3, Rh2, and compound K. Puffing of ginseng has been reported to decrease the content of major ginsenosides and increase that of minor ginsenosides, especially Rg3 in *P. ginseng* [18,25].

**Figure 3:**
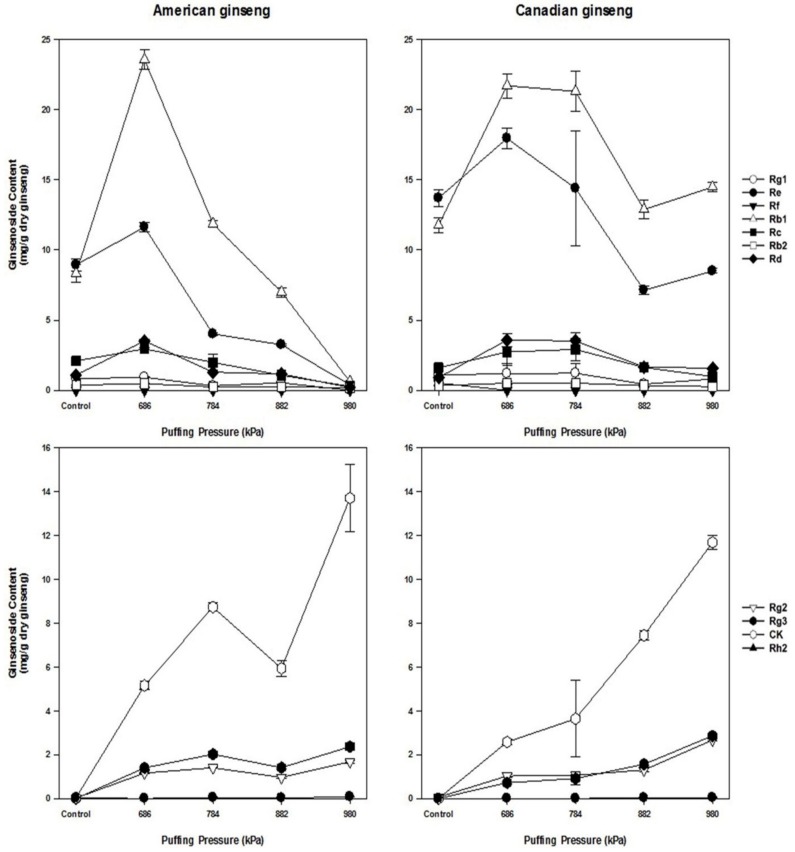
Changes in ginsenoside content of puffed American and Canadian ginsengs.

The changes in the ginsenoside profiles of puffed American and Canadian ginsengs are shown in Figure 3. The content of all major ginsenosides except Rg1 (Rb1, Rb2, Rc, Rd, Re) increased up to 686 kPa and then decreased as the puffing pressure increased in both ginsengs. Decomposition or thermal conversion of ginsenosides occur when heat is applied [34]. The increase in major ginsenoside content at 686 kPa may be attributed to non-soluble polymer components converting into soluble ones due to puffing and to the increased penetration of the extraction solvent due to the porous structure of the tissue [23]. Another possible explanation is the weakening of the binding force due to the destruction of cell walls and to changes in the molecular structure induced by puffing [18,35]. In this study, ginsenosides Rg2, Rg3, Compound K, and Rh2 were newly produced by deglycosylation and pyrolysis of major ginsenosides. Their concentrations increased with increasing puffing pressure. Especially, Compound K concentration greatly increased at 980 kPa compared to the control group in both puffed ginsengs. The contents of ginsenosides Rg2 and Rg3 also significantly increased in both puffed ginsengs. This result is slightly different from previous results on puffed ginseng [25] and puffed red ginseng [18], possibly because different ginseng varieties were used. *P. quinquefolius* has a different ginsenoside profile compared to *P. ginseng*, resulting in different ginsenoside products after puffing. Unlike *P. ginseng,* the levels of ginsenoside F1 and F2 of *P. quinquefolius* were relatively high and did not change with puffing, indicating that those ginsenosides are heat-resistant and not easily transformed by puffing.

### 3.4. Antioxidant Activity, TPC, and TFC

Antioxidant activities (DPPH and ABTS), TPC, and TFC of puffed American and Canadian ginsengs are shown in Table 2. In the control group, no significant differences (*p* < 0.05) in antioxidant activities were observed between American and Canadian ginsengs. Puffed American and Canadian ginsengs showed much higher antioxidant activities, TPC, and TFC compared to the control group. In DPPH, puffing increased the activity approximately 5–6 times compared to the control group, regardless of the puffing pressure, in both American and Canadian ginsengs. ABTS, TPC, and TFC but not DPPH increased with increasing puffing pressure. ABTS radical scavenging activity increased more than 10 times compared with the control group. Especially, at 980 kPa, ABTS of both ginsengs increased approximately 40 times over that of the control group. TPC and TFC in both American and Canadian ginsengs also showed the highest levels at 980 kPa, approximately 20 and 10 times higher than those of the control group, respectively. Water-soluble substances that affect the antioxidant activity exist in an insoluble state in combination with other substances. The increases of antioxidant activities, TPC, and TFC may be explained by the weakening of the molecular binding caused by heat treatment and elution of those materials into a water-soluble state [36] and the increase of certain substances such as maltol, a Maillard reaction product [15]. TPC [23] and TFC [37,38] have been reported to increase with increasing temperature and heat treatment time. Likewise, in this study, antioxidant activities, TPC, and TFC of American and Canadian ginsengs increased during puffing with increasing temperature and holding time.

### 3.5. Acidic Polysaccharides and MRPs

The changes in acidic polysaccharides content of puffed American and Canadian ginsengs are shown in Table 2. Before puffing, American and Canadian ginsengs showed similar acidic polysaccharides contents. However, after puffing, the content of acidic polysaccharides of both ginsengs increased significantly (*p* < 0.05) compared to those of the non-puffed counterparts; the highest levels of acidic polysaccharides resulted from puffing at 980 kPa. No significant differences (*p* < 0.05) were observed by puffing at 686, 784, or 882 kPa in both American and Canadian ginseng. Ginseng contains 60–70% (dry basis) of carbohydrates, the major components of which are pectin and starch [39]. The acidic polysaccharides of ginseng consist of a pectin-like substance with a molecular weight of 34,600 Da, whose main component is galacturonic acid [22]. The majority of the polysaccharides from *P. quinquefolius* are in the neutral form, and their acidic form increases with thermal processing. The content of acidic polysaccharides of ginseng tends to increase with heat treatment [22,32]. Moreover, acidic polysaccharides are affected by temperature rather than heat treatment time [22]. Depending on the specific characteristics of puffing, the greatest concentration of acidic polysaccharides in this study was produced by puffing at 980 kPa in both American and Canadian ginsengs.

The MRPs of puffed American and Canadian ginsengs are shown in Table 2. The MRPs of both American and Canadian ginsengs before puffing were not significantly different (*p* < 0.05) and gradually increased with increasing puffing pressure. The increase in MRPs with thermal processing of American ginseng has been reported [15], and those changes were correlated with changes in antioxidant activity, TPC, and TFC [23,37,38].

### 3.6. Relationships between Antioxidant Activities, TPC, TFC, MRPs, and Acidic Polysaccharides

The results of the Pearson correlations among antioxidant activities, TPC, TFC, amount of MRPs, and acidic polysaccharides content of puffed American and Canadian ginsengs are shown in Table 3. In American ginseng, ABTS correlated well with acidic polysaccharides content, TPC, and amount of MRPs. TFC was in good correlation with TPC and amount of MRPs. TPC and amount of MRPs had good correlation with all other parameters except for DPPH. Moreover, acidic polysaccharides content had a good correlation with amount of MRPs, ABTS, and TPC. In contrast, the correlations for Canadian ginseng were slightly different from those of American ginseng; ABTS correlated well with acidic polysaccharides content, TPC, and TFC. The amount of MRPS had a good correlation with TPC and TFC. TPC and TFC h ad good correlation with all other parameters except for DPPH. Likewise, acidic polysaccharides content had a good correlation with ABTS and TFC.

Although the correlation patterns of American and Canadian ginsengs were not the same, two common correlations were observed. First, acidic polysaccharides content showed the strongest correlation with ABTS in both American and Canadian ginsengs. Second, DPPH did not correlate well with any other antioxidant parameter in either American or Canadian ginseng. These results suggest that compounds and mechanisms related to ABTS are very closely related to acidic polysaccharides content, and that DPPH has distinctive characteristics, different from the other antioxidant parameters evaluated in this study. It is interesting to note that this result is not casual, and this difference may come from the different sources of the samples, their different reactivity when subjected to puffing, and so on. Further analysis is needed for clarifying this result.

## 4. Conclusions

Puffing changed the structure of ginseng from dense to porous leading to easy elution of bioactive compounds from ginseng. This phenomenon increased the extraction yields and crude saponin content of both puffed American and Canadian ginsengs. It is notable that the levels of the major ginsenosides (Rb1, Rb2, Rc, Rd, Re) increased by puffing at 686 kPa, possibly as a result of the formation of a porous structure without thermal conversion of the ginsenosides. Deglycosylation and pyrolysis of major ginsenosides during the puffing process induced the production and increase of minor ginsenosides (Rg2, Rg3, Compound K, and Rh2). Especially, the level of compound K dramatically increased by puffing at 980 kPa in both American and Canadian ginsengs. Moreover, puffing greatly increased antioxidant activities (ABTS and DPPH), and the increase of MRPs level seemed to significantly affect the ABTS of puffed ginsengs. Puffed American and Canadian ginsengs showed higher antioxidant activities and higher minor ginsenoside levels than non-puffed ginsengs, a trend similar to that of puffed *P. ginseng*. There was no significant difference between American and Canadian ginsengs in either control or puffed samples. In the case of *P. ginseng,* red ginseng, and black ginseng, the preparation takes a very long time, which is not economical. On the other hand, the preparation of puffed ginseng takes relatively a very short time, and more effective changes in ginsenosides and bioactive compounds can be obtained. Therefore, puffing is an efficient and economic processing method appropriate for both American and Canadian ginsengs to enhance ginseng’s antioxidant activity and produce minor ginsenosides, such as Rg3, Compound K, and Rh2.

## Figures and Tables

**Table 1:** Color, extraction yield, and crude saponin content of puffed American and Canadian ginsengs.

Sample (kPa)	L	a	b	Extraction Yield (%)	Crude Saponin Content (mg/g Dried Ginseng)
A-Control	76.86 ± 1.55 ^a,^*	8.34 ± 0.15 ^d^	16.99 ± 0.53 ^d^	35.03 ± 8.49 ^d^	72.44 ± 5.19 ^e^
A-686	54.66 ± 10.14 ^b^	12.91 ± 1.15 ^ab^	27.64 ± 2.21 ^ab^	46.86 ± 12.07 ^ab^	141.20 ± 26.16 ^a^
A-784	47.56 ± 14.73 ^bc^	12.20 ± 1.00 ^bc^	25.97 ± 3.90 ^abc^	44.84 ± 5.74 ^b^	126.56 ± 12.26 ^abc^
A-882	44.87 ± 10.28 ^bc^	11.73 ± 2.29 ^bc^	24.55 ± 5.16 ^bc^	40.44 ± 4.16 ^c^	107.12 ± 17.39 ^d^
A-980	39.44 ± 9.58 ^c^	10.78 ± 2.51 ^c^	21.52 ± 6.24 ^c^	46.19 ± 3.20 ^ab^	122.10 ± 14.78 ^bcd^
C-Control	78.67 ± 1.92 ^a^	8.41 ± 0.28 ^d^	16.77 ± 1.04 ^d^	38.57 ± 3.92 ^cd^	104.33 ± 22.08 ^d^
C-686	54.26 ± 4.08 ^b^	14.27 ± 0.50 ^a^	30.56 ± 1.02 ^a^	46.95 ± 4.43 ^ab^	129.44 ± 17.68 ^ab^
C-784	53.53 ± 7.51 ^b^	13.28 ± 1.05 ^ab^	28.69 ± 2.36 ^ab^	47.32 ± 3.54 ^ab^	115.86 ± 13.42 ^bcd^
C-882	48.04 ± 3.86 ^b^	13.41 ± 1.30 ^ab^	27.85 ± 2.55 ^ab^	49.44 ± 5.26 ^a^	109.61 ± 9.29 ^cd^
C-980	39.82 ± 2.42 ^c^	11.94 ± 1.24 ^bc^	23.86 ± 2.68 ^bc^	49.20 ± 5.04 ^a^	126.33 ± 10.66 ^abc^

A, American ginseng; C, Canadian ginseng. * Values with the same letter in the column are not significantly different *(p <* 0.05).

**Table 2:** Antioxidant activity (2,2-diphenyl-1-picrylhydrazyl (DPPH) and 2,2′-azino-bis (3-ethylbenzothiazoline-6-sulphonic acid (ABTS) radical scavenging activities), TPC, TFC, acidic polysaccharides, and Maillard reaction products (MRPs) of puffed American and Canadian ginsengs.

Sample (kPa)	DPPH (mg VCE/ g Dried Ginseng)	ABTS (mg VCE/ g Dried Ginseng)	TPC (mg GAE/ g Dried Ginseng)	TFC (mg CE/ g Dried Ginseng)	AP (mg GA/ g Dried Ginseng)	MRPs
A-Control	0.29 ± 0.11 ^b*^	0.97 ± 0.15 ^d^	1.11 ± 0.06 ^f^	0.44 ± 0.05 ^e^	1.78 ± 0.27 ^c^	0.186 ± 0.014 ^c^
A-686	1.88 ± 0.26 ^a^	10.06 ± 4.88 ^c^	9.24 ± 3.60 ^e^	2.39 ± 1.17 ^de^	2.60 ± 0.61 ^bc^	0.295 ± 0.085 ^bc^
A-784	1.97 ± 0.16 ^a^	14.58 ± 5.23 ^bc^	14.46 ± 5.05 ^cde^	3.84 ± 1.66 ^bcd^	2.87 ± 0.37 ^b^	0.422 ± 0.129 ^b^
A-882	1.89 ± 0.28 ^a^	15.37 ± 3.99 ^bc^	16.05 ± 4.80 ^cd^	4.82 ± 2.15 ^abc^	2.81 ± 0.10 ^bc^	0.442 ± 0.033 ^b^
A-980	1.67 ± 0.33 ^a^	39.71 ± 1.02 ^a^	24.23 ± 5.89 ^a^	5.73 ± 2.11 ^ab^	5.09 ± 1.21 ^a^	0.694 ± 0.209 ^a^
C-Control	0.41 ± 0.20 ^b^	1.24 ± 0.19 ^d^	1.65 ± 0.22 ^f^	0.56 ± 0.06 ^e^	1.79 ± 0.42 ^c^	0.215 ± 0.043 ^c^
C-686	2.04 ± 0.15 ^a^	13.04 ± 3.70 ^bc^	12.51 ± 4.69 ^de^	3.54 ± 1.04 ^cd^	3.11 ± 0.39 ^b^	0.317 ±0.075 ^bc^
C-784	1.94 ± 0.18 ^a^	17.58 ± 7.58 ^b^	12.87 ± 2.39 ^cde^	4.20 ± 1.41 ^abcd^	3.02 ± 0.15 ^b^	0.400 ± 0.140 ^b^
C-882	1.74 ± 0.22 ^a^	17.28 ± 0.34 ^b^	18.48 ± 3.02 ^bc^	4.91 ± 1.73 ^abc^	3.25 ± 0.45 ^b^	0.616 ± 0.146 ^a^
C-980	1.85 ± 0.23 ^a^	39.64 ± 3.13 ^a^	21.33 ± 6.21 ^ab^	6.19 ± 1.34 ^a^	4.69 ± 0.69 ^a^	0.645 ± 0.204 ^a^

A, American ginseng; C, Canadian ginseng; DPPH, DPPH radical scavenging activity; ABTS, ABTS radical scavenging activity; TPC, total phenolic content; TFC, total flavonoid content; AP, acidic polysaccharides; VCE: vitamin C equivalent; GAE: gallic acid equivalent; CE: catechin equivalent; GA, galacturonic acid; MRPs, Maillard reaction products. ^*^ Values with the same letter in the column are not significantly different (*p* < 0.05).

**Table 3:** Pearson correlations between amount of MRPs, DPPH, ABTS, TPC, TFC, and acidic polysaccharides content of puffed American and Canadian ginsengs.

American Ginseng	MRPs	DPPH	ABTS	TPC	TFC	AP
MRPs						
DPPH	0.548					
ABTS	0.982 **	0.471				
TPC	0.981 **	0.693	0.942 *			
TFC	0.938 *	0.737	0.868	0.981 **		
AP	0.965 **	0.444	0.997 ***	0.918 *	0.831	
**Canadian Ginseng**						
MRPs						
DPPH	0.548					
ABTS	0.835	0.613				
TPC	0.937 *	0.793	0.88 **			
TFC	0.910 *	0.820	0.911 *	0.989 **		
AP	0.842	0.705	0.982 **	0.926 *	0.941 *	

MRPs, Maillard reaction products; DPPH, DPPH radical scavenging activity; ABTS, ABTS radical scavenging activity; TPC, total phenolic content; TFC, total flavonoid content; AP, acidic polysaccharides. * indicates *p* < 0.05; ** indicates *p* < 0.01; *** indicates *p* < 0.001.
